# Supplementary figures and images for: Population genetic diversity of Schistosoma japonicum arises from the host switching in the life cycle
Source: PLoS Negl Trop Dis. 2025 Mar 19;19(3):e0012931. doi: 10.1371/journal.pntd.0012931 (PMC11949366; doi:10.1371/journal.pntd.0012931)

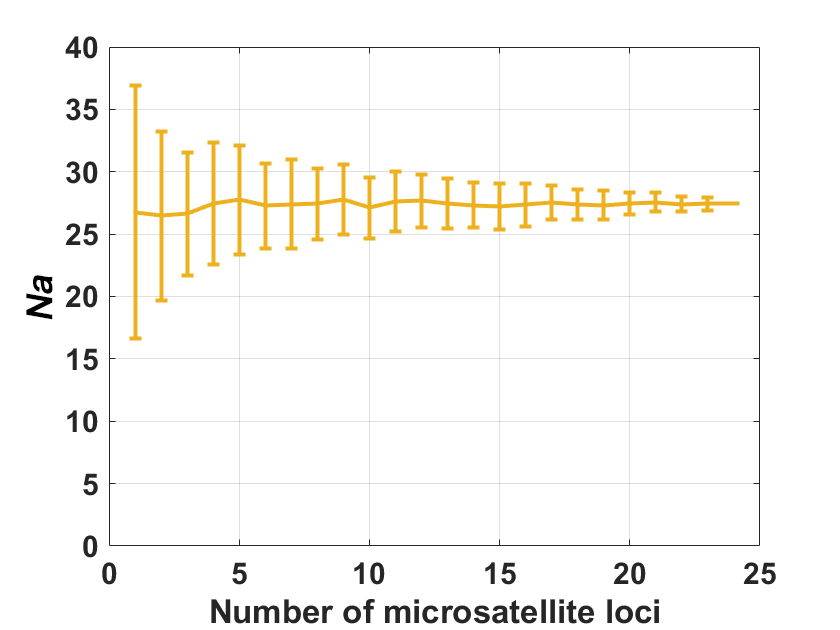

Supplement: S1 Fig — The number of microsatellite markers affects the deviation of genetic diversity of cercariae. The curve in the middle represents the mean value of Na for the different number of loci. The standard deviation of Na among replicates progressively decreased with the increase in the number of loci. (TIF) [file pntd.0012931.s008.tif]
