# Supplementary material for: Population genetic diversity of Schistosoma japonicum arises from the host switching in the life cycle
Source: PLoS Negl Trop Dis. 2025 Mar 19;19(3):e0012931. doi: 10.1371/journal.pntd.0012931 (PMC11949366; doi:10.1371/journal.pntd.0012931)
Supplement: Attachment — Submitted filename: Response to Reviewers.docx [file pntd.0012931.s010.docx]

Dear respected editors:

Thank you for the comments and for allowing us to revise the manuscript. All authors appreciate it very much and revised the manuscript according to your advice. All comments are responded point by point, and details are listed below. The alternations in the manuscript were marked and highlighted in the tracked changes mode. The new version of the manuscript was double-checked, and we hope it meets the PLoS Neglected Tropical Diseases's publication criteria with better quality.

**Additional Editor Comments:**

The manuscript investigated the genetic diversity of Schistosoma japonicum caused by host switching during it's life cycle.

The topic of the manuscript is very interesting, however, the information in the manuscript is scattered, especially in the background section, for example: lines 74-76 give an impression that Schistosoma japonicum main hosts are snails and humans, although it was mentioned somewhere else that it can infect snails and mammals in general, nevertheless based it's results on studying microsatellites markers in population covering non-mammalian organisms (with the exception of human), those organisms were plants, fruit flies, fish and sponges (line 599)

My recommendation is that the manuscript needs to be re-organized, and clarification of why using microsatellite markers based on organisms that are not the main host of Schistosoma japonicum, ie. plants, fruit flies, fish and sponges.

**Reply:** Thanks a lot for the valuable comments and the recommendation.

And we feel sorry for the confusing description, which caused the misunderstanding.

You are correct that non-mammalian organisms, including plants, fruit flies, fish and sponges, are not the definitive hosts of *Schistosoma japonicum*. We mentioned these referenced organisms to introduce the phenomenon that variable numbers of microsatellite markers were used in related population genetics studies, which may finally lead to inconsistent results even for the same species of organism. That is also why we discussed the number of microsatellite markers used in this study, which was additionally analyzed and confirmed as described in the discussion and had never been discussed for *S. japonicum* studies before.

Thanks again for your advice. We re-organized the description in the introduction related to the hosts of *S. japonicum* and rewrote the sentences related to the hosts and microsatellite markers in the discussion. Please check all the details that have been changed and highlighted in the manuscript, which are also listed below.

Minor changes we made in the text with tracked changes are listed as follows:

1. Line 69-85: the Author summary was added.

2. Line 87: "Background" was changed into "Introduction".

3. Line 92-93: "……in the definitive mammalian hosts. Humans and other definitive hosts were infected……" was changed into "……in the definitive hosts, including humans and some mammalians. Definitive hosts were infected……".

4. Line 127: "can vary" was changed to "were proved to vary"

5. Line 139: "include more than 40….." was changed to "include humans and more than 40……"

6. Line 165: "like mice and rabbits" was added.

7. Line 190, 194, and 225: ^TM^ were deleted.

8. Line 364: "worms" was added.

9. Line 510: "from different sampling sizes of mice and rabbit" was changed to "from different mice and rabbits sampling sizes".

10. Line551: "the population of cercariae" was changed to "the cercariae population".

11. Line553: "the development in different stages" was changed to "different developmental stages

12. Line 590, line 611: "the" s were added.

13. Line 628-630: "The microsatellite markers numbers …… sponges" was changed to "In previous related population structure studies, the number of microsatellite markers had been widely evaluated in several organisms, such as human…sponges".

14. Line 635: "organisms including" was added.

15. Line 636: "of *S. japonicum*" was added.

16. Line 656: "Herein" was deleted and changed to "Additionally".

17. Line 660: "data not shown here" was deleted.

…

All of the points in the Journal Requirements were revised in the manuscript according to the journal's criteria, and the responses were not described by points as requested, but all changes can be traced in the manuscript and listed above.

Figure files were uploaded to the Preflight Analysis and Conversion Engine (PACE) and converted before resubmission.

Once again, thank you very much!

Best wishes!

Sincerely

Qin-Ping Zhao
